# PReS13-SPK-1127: Pediatric rheumatology in India

**DOI:** 10.1186/1546-0096-11-S2-I34

**Published:** 2013-12-05

**Authors:** RP Khubchandani

**Affiliations:** 1 Pediatrics, Jaslok Hospital And Research Center, Mumbai, India

## 

Pediatric Rheumatology took birth in India with the formation of an interest group of the Indian Academy of Pediatrics and the first conference in 2001 in Mumbai. Now 11 conferences old, it has about 200 members and 12 centers offering part time or whole time care.

With a population of over 1000 million, it may be extrapolated from Western data that India should have at least 400000 children with rheumatologic disease - JIA, JDM, SLE and vasculitides (excluding KD and HSP) Disease patterns are similar to a Western clinic and a little over a third of children attending a rheumatology clinic in Mumbai are diagnosed to have non rheumatologic musculoskeletal disease, notably leukemia, genetic disorders and chronic pain syndromes. The term 'musculoskeletal medicine clinic' seems more apt. Acute diseases such as septic arthritis/osteomyelitis are probably filtered at the primary care level and hence the clinic functions mainly as a chronic or day care facility. Fluorosis, musculoskeletal MDR tuberculosis, brucellosis and HIV associated arthropathy are unique issues. Most centers report declining trends of Rheumatic fever but an alarming increase of KD with consequent IViG requirement. Rare diseases such as hereditary fever syndromes are seen, some possibly representing the vestiges of early European colonization.

Individual trends differ with SOJIA and ERA forming the bulk of JIA rather than OJIA. Uveitis is not a major concern. Outcomes of individual diseases seem different to the West with most centers in India reporting a milder phenotype of JDM.

Paradoxically 80% of the world's pediatric rheumatologists care for 20% of the world's children and vice versa. Our undergraduate teaching ignores rheumatology while our post graduate teaching focuses more on acute cross sectional care rather than chronic longitudinal care.

Patents usually reach through complex pathways of referral, often having consulted adult orthopaedic surgeons or alternative medicine systems prior to their visit, highlighting the need for increasing awareness about our specialty to lay public. They often have to travel 300-500 km for a consultation. Consequently, delay in diagnosis of various diseases, irregular follow up and frequent usage of steroid prior to referral are common. While raising money for one time use of IViG is still feasible (selfpaid or charity), a country with a per capita income of 1219 USD and much larger problems is unable to adapt treatment guidelines suggested by the West when it comes to the use of biologicals and consequently lesser studied drugs and treatment algorithms are resorted to.

The crunch for interested and trained personnel (at least 200) has been addressed through a 3 level process -Preach -sensitizing large gatherings of pediatricians, Reach and Teach-intensive weeklong courses to committed smaller groups and Each to Each -one on one teaching through fellowships. The results are slow but visible.

India can contribute to the world of pediatric rheumatology through its sheer numbers and its English speaking medical community thus providing an excellent collaborative research and teaching opportunity to centers with knowhow but lacking in numbers. The developed world can help us through facilitating student exchange, concessional diagnostic tests and courses, telemedicine, bulletin board, Asian editions of books or open access publications and patient information.

## Disclosure of interest

None declared.

